# In-silico phenotype prediction by normal mode variant analysis in *TUBB4A*-related disease

**DOI:** 10.1038/s41598-021-04337-x

**Published:** 2022-01-07

**Authors:** Avi Fellner, Yael Goldberg, Dorit Lev, Lina Basel-Salmon, Oded Shor, Felix Benninger

**Affiliations:** 1 Raphael Recanati Genetics Institute, Rabin Medical Center, Beilinson Hospital, 49100 Petah Tikva, Israel; 2 Department of Neurology, Rabin Medical Center, Beilinson Hospital, 49100 Petah Tikva, Israel; 3 Sackler Faculty of Medicine, Tel-Aviv University, 69978 Tel-Aviv, Israel; 4 Metabolic-Neurogenetic Clinic, Wolfson Medical Center, 58220 Holon, Israel; 5 Rina Mor Institute of Medical Genetics, Wolfson Medical Center, 58220 Holon, Israel; 6 Felsenstein Medical Research Center, 49100 Petah Tikva, Israel

**Keywords:** Disease genetics, Clinical genetics, Neurological disorders, Molecular modelling, Protein function predictions, Computational models

## Abstract

*TUBB4A*-associated disorder is a rare condition affecting the central nervous system. It displays a wide phenotypic spectrum, ranging from isolated late-onset torsion dystonia to a severe early-onset disease with developmental delay, neurological deficits, and atrophy of the basal ganglia and cerebellum, therefore complicating variant interpretation and phenotype prediction in patients carrying *TUBB4A* variants. We applied entropy-based normal mode analysis (NMA) to investigate genotype–phenotype correlations in *TUBB4A*-releated disease and to develop an in-silico approach to assist in variant interpretation and phenotype prediction in this disorder. Variants included in our analysis were those reported prior to the conclusion of data collection for this study in October 2019. All *TUBB4A* pathogenic missense variants reported in ClinVar and Pubmed, for which associated clinical information was available, and all benign/likely benign *TUBB4A* missense variants reported in ClinVar, were included in the analysis. Pathogenic variants were divided into five phenotypic subgroups. In-silico point mutagenesis in the wild-type modeled protein structure was performed for each variant. Wild-type and mutated structures were analyzed by coarse-grained NMA to quantify protein stability as entropy difference value (ΔG) for each variant. Pairwise ΔG differences between all variant pairs in each structural cluster were calculated and clustered into dendrograms. Our search yielded 41 *TUBB4A* pathogenic variants in 126 patients, divided into 11 partially overlapping structural clusters across the TUBB4A protein. ΔG-based cluster analysis of the NMA results revealed a continuum of genotype–phenotype correlation across each structural cluster, as well as in transition areas of partially overlapping structural clusters. Benign/likely benign variants were integrated into the genotype–phenotype continuum as expected and were clearly separated from pathogenic variants. We conclude that our results support the incorporation of the NMA-based approach used in this study in the interpretation of variant pathogenicity and phenotype prediction in *TUBB4A*-related disease. Moreover, our results suggest that NMA may be of value in variant interpretation in additional monogenic conditions.

## Introduction

Hypomyelinating leukodystrophies comprise a heterogeneous group of hereditary disorders, characterized by abnormal myelin development and typical brain MRI findings^1,2^. Of these disorders, the ones associated with heterozygous pathogenic variants in *TUBB4A* demonstrate a broad phenotypic spectrum, including primary dystonia (DYT4; Autosomal dominant torsion dystonia-4; MIM#128101), isolated hypomyelination, hypomyelination with atrophy of the basal ganglia and cerebellum (H-ABC; Hypomyelinating leukodystrophy 6; MIM#612438), as well as early infantile encephalopathy^2^.

*TUBB4A* encodes Tubulin beta-4A protein, which constitutes a principal part of microtubules, together with Tubulin-alpha^3^. It is expressed predominantly in the CNS, especially in the cerebellum, white matter and putamen^4^. Over 40 disease-associated *TUBB4A* pathogenic variants have been identified, all of which are missense variants (Hamilton et al.^3^ and ClinVar 2019 (https://www.ncbi.nlm.nih.gov/clinvar)). Previous reports have suggested a possible genotype–phenotype correlation in *TUBB4A*-related disease. At the milder end of the phenotypic spectrum the p.Arg2Gly and p.Ala271Thr variants are associated with adult-onset DYT4 dystonia^4,5^. At the most severe end of this spectrum the p.Asn414Lys variant is associated with infantile-onset encephalopathy and early death^2,6^. H-ABC patients with the most common *TUBB4A* disease-associated variant (p.Asp249Asn) have a less severe clinical course compared to other H-ABC presentations associated with this gene^2,3^. Moreover, several *TUBB4A* pathogenic variants result in hypomyelination with no atrophy of the basal ganglia.

Interpretation of the effect of genetic variants at the protein level can be assisted by computational approaches. Over the last two decades, many in silico tools were developed for that end and were used to investigate the effects of gene variants in human disease^7–11^. Despite the multitude of computational tools available, only a few newer methods exploit the 3D protein structure to include dynamic aspects of proteins in the prediction of genetic variants impact^7^. Normal mode analysis (NMA) is one of the main approaches that enable the incorporation of dynamic parameters of protein structure into the investigation of genetic variants^7^. In this study, we utilize an in-silico approach using NMA to investigate the effect of disease-associated *TUBB4A* missense variants on the encoded protein and their associated phenotype. This approach can be used in the classification of known missense variants and possibly predict the effect and phenotype of new variants of uncertain significance (VUS).

## Subjects and methods

### *TUBB4A* variants included in the analysis

We performed a search in ClinVar and in Pubmed for reported disease-associated *TUBB4A* variants. This search was concluded in October 2019. We included in the analysis all disease-associated variants reported, for which associated clinical phenotypic information was available. Cases of mosaicism in affected parents were not included in the analysis. Pathogenic missense variants were included in the analysis, as well as benign and likely benign *TUBB4A* missense variants reported in ClinVar prior to the conclusion of data collection for this study in October 2019. In addition, we searched the GnomAD database for common *TUBB4A* missense variants in the general population in order to include them in the analysis as well.

### Clinical phenotype distribution

We subdivided the included patients into five subgroups according to their clinical phenotype: DYT4 dystonia (group-1), H-ABC with a milder course associated with the p.Asp249Asn variant (m-H-ABC; group-2), hypomyelination with no basal ganglia atrophy (HM; group-3), classical H-ABC (c-H-ABC; group-4) and early infantile encephalopathy associated with the p.Asn414Lys variant (EIE; group-5). Group-3 was further subdivided into group-3a that includes cases with infantile disease onset and severe progressive motor impairment, and group-3b that includes patients who had a prolonged, less severe clinical course following either a later disease onset in teenage years or an infantile/childhood disease onset with achievement of unassisted walking.

### In-silico analysis

The structure of the Tubulin-Stathmin-like domain complex was taken from the Protein Data Bank (PDB-101; accession numbers PDB: 1FFX)^12^. The crystal structure of the A and C chains is identical to the Tubulin alpha-1A chain of Sus scrofa (P02550-TBA1A_PIG). Their amino-acid sequence is identical with the human Tubulin alpha-1A chain and we considered them as homologous to the human Tubulin alpha-1A structure and retained them unchanged in our model structure. The B and D chains of the original structure are identical with the tubulin beta chain (P02554-TBB_PIG, from Sus scrofa) and we modified the B and D chain amino-acid sequences to be identical to the human TUBB4A as follows: We aligned the amino acid sequence of the human TUBB4A protein and chains B and D from TBB_PIG using BLAST (LASTP suite) resulting in 97.19% similarity^13,14^. Next, using the mutagenesis plugin in PyMol Molecular Graphics System Version 1.8 (Schrödinger, LLC., Cambridge, MA) we performed in-silico mutagenesis of chains B and D of the original sequence (TBB_PIG) to replace non-identical amino acids with those of the human TUBB4A to obtain 100% identity with the human TUBB4A protein. It should be noted that homology modeling (for example by the Swiss model server) produces an over-optimized protein structure lacking the original 1FFX structure characteristics. This over-optimization is clearly demonstrated in the Ramachandran plot analysis when the homology modeling is compared to the original protein structure and to the non-optimized structure produced by the PyMol mutagenesis approach used in this study (Supplementary Fig. 1). Therefore, the PyMol mutagenesis structure approach was used in order to retain the characteristics of the original structure.

**Figure 1:**
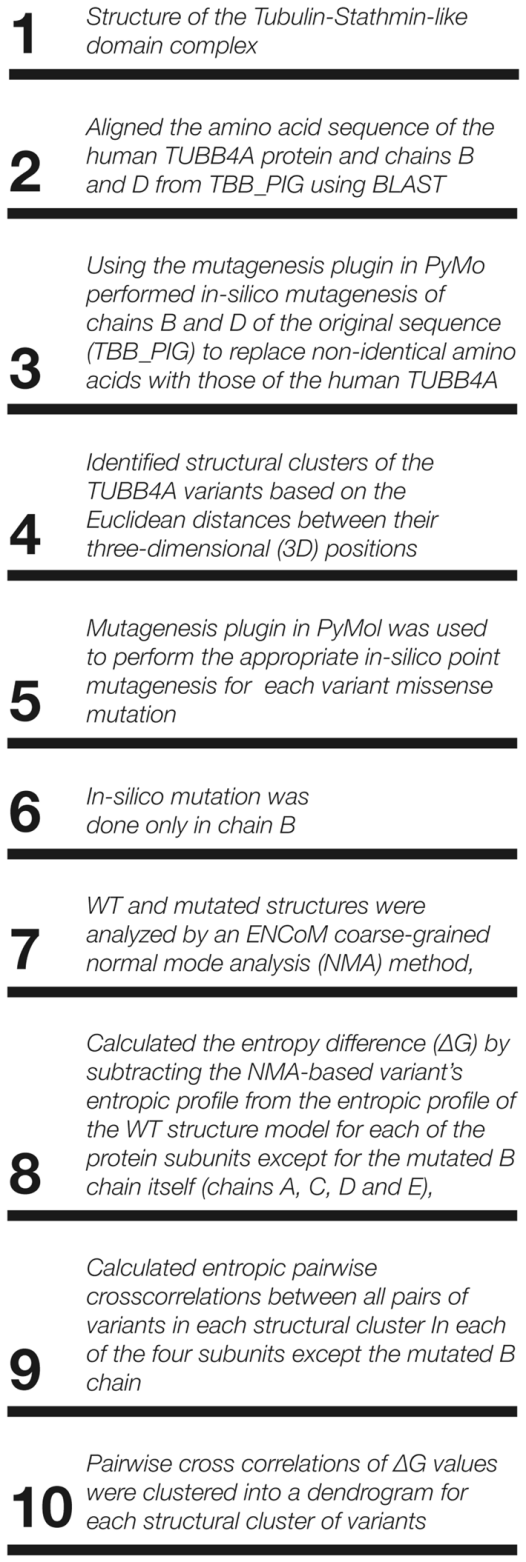
Methodology summary. A step-by-step summary of the methodology used to investigate *TUBB4A* variants with normal mode analysis.

We identified structural clusters of the *TUBB4A* variants based on the Euclidean distances between their three-dimensional (3D) positions. Clusters were defined based on a mean distance of less than 15 Å between all variants in a cluster and the cluster’s center of mass. Mutagenesis plugin in PyMol Molecular Graphics System Version 1.8 (Schrödinger, LLC., Cambridge, MA) was used to perform the appropriate in-silico point mutagenesis in the wild-type (WT) modeled structure. Using this structure, in-silico mutagenesis was performed for each variant to replace the amino acid in the corresponding position. In-silico mutation was done only in chain B, in order to comply with an autosomal dominant (heterozygous) heredity pattern. WT and mutated structures were analyzed by an ENCoM coarse-grained normal mode analysis (NMA) method, in order to evaluate the effect of the analyzed variants on the stability of the protein. This method is based on an entropic considerations C package of ENCoM^15^ available at ENCoM development website. This package is compiled and used on an Ubuntu platform (Canonical Group, UK). For each analyzed variant, we calculated the entropy difference (ΔG) by subtracting the NMA-based variant’s entropic profile from the entropic profile of the WT structure model. This was done for each of the protein subunits except for the mutated B chain itself (chains A, C, D and E), as well as for the GTP bound to the protein (represented by subunit K). The calculation of the entropic difference (∆G) was done using MATLAB software (Mathworks, Natick, MA). Entropic ΔG pairwise cross correlation between all pairs of variants in each structural cluster was calculated for each of the four protein subunits, except for the mutated B chain itself. These pairwise cross correlations of ΔG values were clustered into a dendrogram for each structural cluster of variants. Dendrograms were computed by the Ward algorithm with MATLAB software (Mathworks, Natick, MA). Our methodology is summarized in Fig. 1.

### Standard protocol approvals, registrations, and patient consents

This study was based on in-silico analysis of known genetic variants from public databases and the literature and did not involve patients. Therefore, it did not require an ethical committee approval or informed consent.

## Results

### Reported disease-associated *TUBB4A* variants

Our ClinVar and Pubmed search yielded 41 *TUBB4A* (NM_006087.4) pathogenic variants for which associated clinical information was available, in 126 patients. All pathogenic variants were missense substitutions. These variants and their associated phenotype subgroups are detailed in Table 1. Our search in ClinVar for benign and likely benign missense variants in *TUBB4A* (canonical transcript: NM_006087.4) yielded a single benign variant (c.238C > T p.Pro80Ser) and a single likely benign variant (c.667G > A p.Gly223Arg). These were included in the analysis. GnomAD database does not include polymorphisms (defined as missense variants with frequency ≥ 1.0% in the general population) in the canonical *TUBB4A* transcript (NM_006087.4). Yet, in order to add available data to our analysis, the most common *TUBB4A* missense variant found in the GnomAD browser (NM_006087.4: c.830G > C p.Gly277Ala, 30 carriers, minor allele frequency 0.012%) was included in the analysis as an additional benign variant. Other GnomAD missense variants in the canonical *TUBB4A* gene transcript are significantly rarer (of these, the most common variant, c.1331C > T p.Ala444Val, has a minor allele frequency of 0.005%), and therefore could not be undoubtedly included in the analysis as common benign variants in the general population.

**Table 1:** *TUBB4A* pathogenic variants included in the analysis and their associated phenotypic subgroups.

Group	Variant	Number of patients	References
Group-1 (DYT4 dystonia)	c.4C > G p.Arg2Gly	12	Hersheson et al.^4^ and Lohmann et al.^5^
	c.811G > A p.Ala271Thr	1	Lohmann et al.^5^
Group-2 (m-H-ABC)	c.745G > A p.Asp249Asn	48	Hamilton et al.^3^, Simons et al.^16^, Miyatake et al. et al.^17^, Ferreira et al.^18^, Pizzino et al.^19^, Erro et al.^20^, Tonduti et al.^21^ and Delgado et al.^22^
Group-3a (HM)	c.467G > T p.Arg156Leu	1	Purnell et al.^23^
	c.535G > C p.Val179Leu	1	Isakov et al.^24^
	c.539T > G p.Val180Gly	1	Vanderver et al.^25^
	c.538G > A p.Val180Met	1	Ji et al.^26^
	c.568C > T p.His190Tyr	6	Kancheva et al.^27^ and Nicita et al.^28^
	c.763G > A p.Val255Ile	1	Pizzino et al.^19^
	c.785G > A p.Arg262His	5	Miyatake et al.^17^, Ferreira et al.^18^, Ji et al.^26^, Srivastava et al.^29^ and Shimojima et al.^30^
	c.874C > A p.Gln292Lys	1	Pizzino et al.^19^
	c.900G > T p.Met300Ile	3	Erro et al.^20^ and Pyle et al.^31^
	c.1091C > A p.Ala364Asp	1	Pyle et al.^31^
	c.1172G > A p.Arg391His	1	Pizzino et al.^19^ and Vanderver et al.^25^
Group-3b (HM with a less severe phenotype)	c.286G > A p.Gly96Arg	1	Lu et al.^32^
	c.533C > T p.Thr178Met	1	Tonduti et al.^21^
	c.845G > C p.Arg282Pro	2	Pizzino et al.^19^
	c.937G > T p.Val313Leu	1	Macaron et al.^33^
	c.1064A > T p.Asp355Val	1	Sagnelli et al.^34^
	c.1228G > A p.Glu410Lys	3	Blumkin et al.^35^, Miyatake et al.^17^ and Sasaki et al.^36^
Group-4 (c-H-ABC)	c.4C > T p.Arg2Trp	2	Hamilton et al.^3^
	c.5G > A p.Arg2Gln	2	Hamilton et al.^3^ and Miyatake et al.^17^
	c.533C > G p.Thr178Arg	2	Miyatake et al.^17^ and Joyal et al.^37^
	c.544C > A p.Pro182Thr	1	Tonduti et al.^21^
	c.716G > T p.Cys239Phe	2	Hamilton et al.^3^ and Ferreira et al.^18^
	c.730G > A p.Gly244Ser	4	Hamilton et al.^3^ and Carvalho et al.^38^
	c.731G > T p.Gly244Val	2	Hamilton et al.^3^ and Tonduti et al.^21^
	c.731G > A p.Gly244Asp	3	Tonduti et al.^21^
	c.743C > A p.Ala248Asp	1	Arai-Ichinoi et al.^39^
	c.941C > T p.Ala314Val	1	Erro et al.^20^
	c.968T > G p.Met323Arg	1	Hamilton et al.^3^
	c.1054G > A p. Ala352Thr	2	Hamilton et al.^3^
	c.1061G > A p.Cys354Tyr	1	Hamilton et al.^3^
	c.1099T > C p.Phe367Leu	1	Hamilton et al.^3^
	c.1099T > A p.Phe367Ile	1	Hamilton et al.^3^
	c.1162A > G p.Met388Val	2	Hamilton et al.^3^ and Miyatake et al.^17^
	c.1163T > C p.Met388Thr	2	Hamilton et al.^3^ and Tonduti et al.^21^
	c.1164G > A p.Met388Ile	1	Hamilton et al.^3^
	c.1181T > G p.Phe394Cys	1	Carvalho et al.^38^
	c.1190G > T p.Trp397Leu	1	Ji et al.^26^
Group-5 (EIE)	c.1242C > G p.Asn414Lys	1	Duncan et al.^6^

*EIE* early infantile encephalopathy, *H-ABC* hypomyelination with atrophy of the basal ganglia and cerebellum (*c-H-ABC* classical, *m-H-ABC* milder course), *HM* hypomyelination with no basal ganglia atrophy.

### NMA optimization through identification of structural clusters of variants

**Figure 2:**
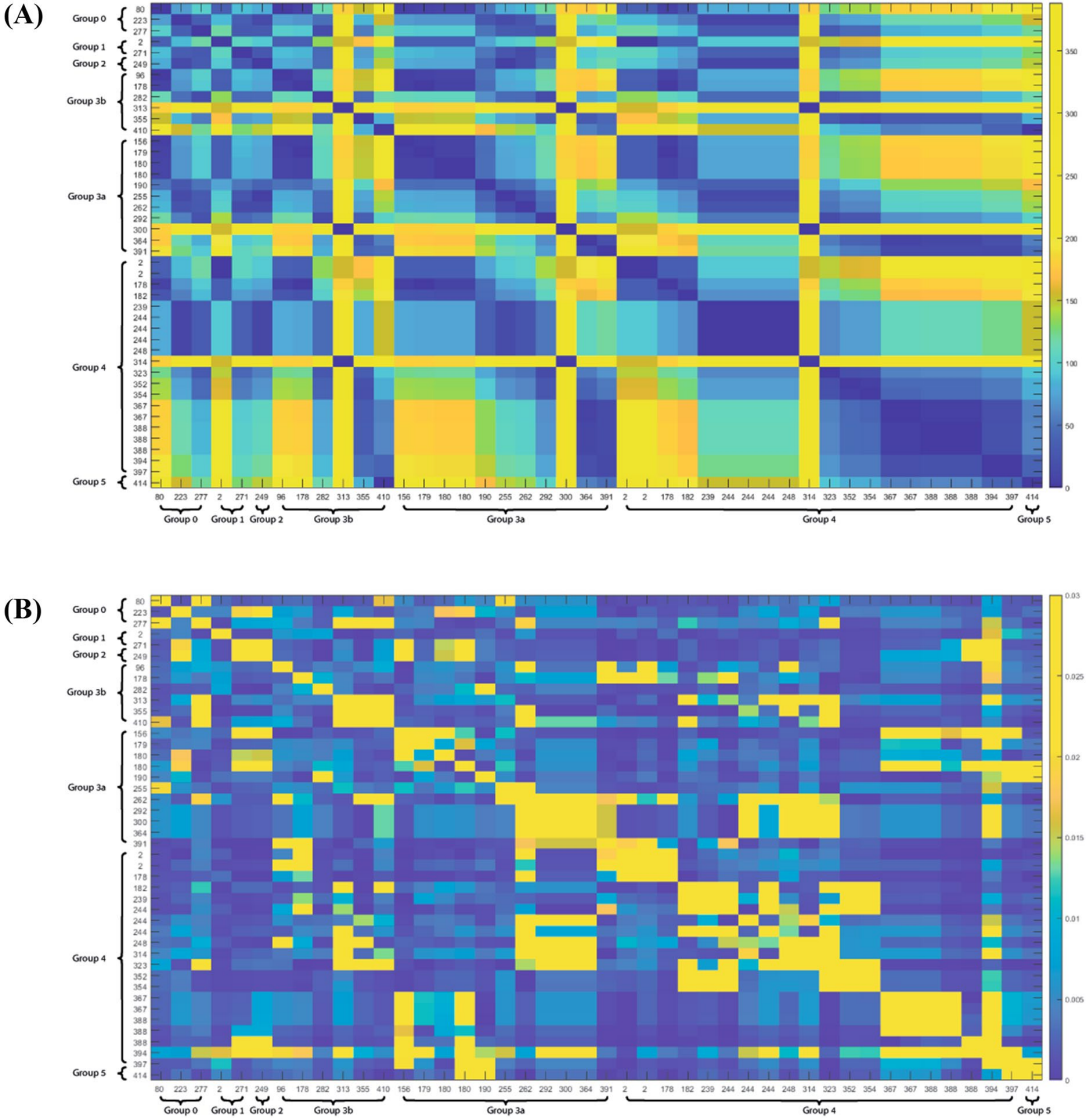
Heatmaps of correlation between phenotype and either variant position or entropic profile. (**A**) Heatmap of pair-wise distances between positions of residues that are mutated in each variant. (**B**) Heatmap of pair-wise cross-correlation coefficient between entropic profile of residues that are mutated in each variant. Order of variants in both heatmaps (**A,B**) is set according to their phenotypic group and in each phenotypic group according to the residue numbers, which are shown across both axes of each heatmap.

**Figure 3:**
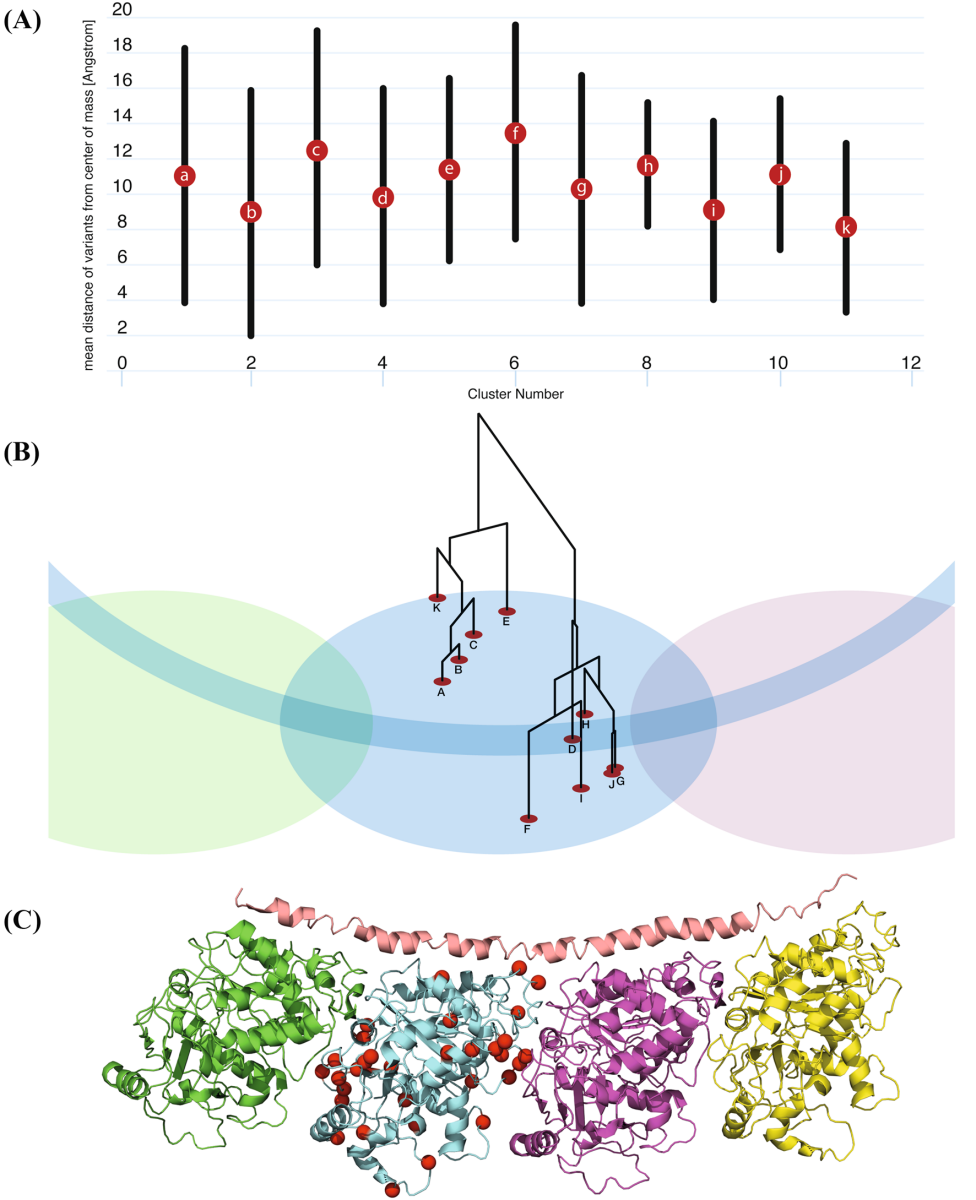
Eleven structural clusters of *TUBB4A* variants. (**A**) Mean distance between all mutated residues in the three-dimensional position in each of the 11 clusters of variants (clusters a–k). (**B**) Schematic representation of the tubulin structure with TUBB4A colored in light blue. The center of mass of each cluster of variants (centers A–K) is depicted by a red circle. Relative distances (in Å) between center of masses are represented by a dendrogram. (**C**) Structure of the 1FFX Tubulin:Stathmin-like domain complex. The positions of the analyzed *TUBB4A* variants are labeled in red.

**Figure 4:**
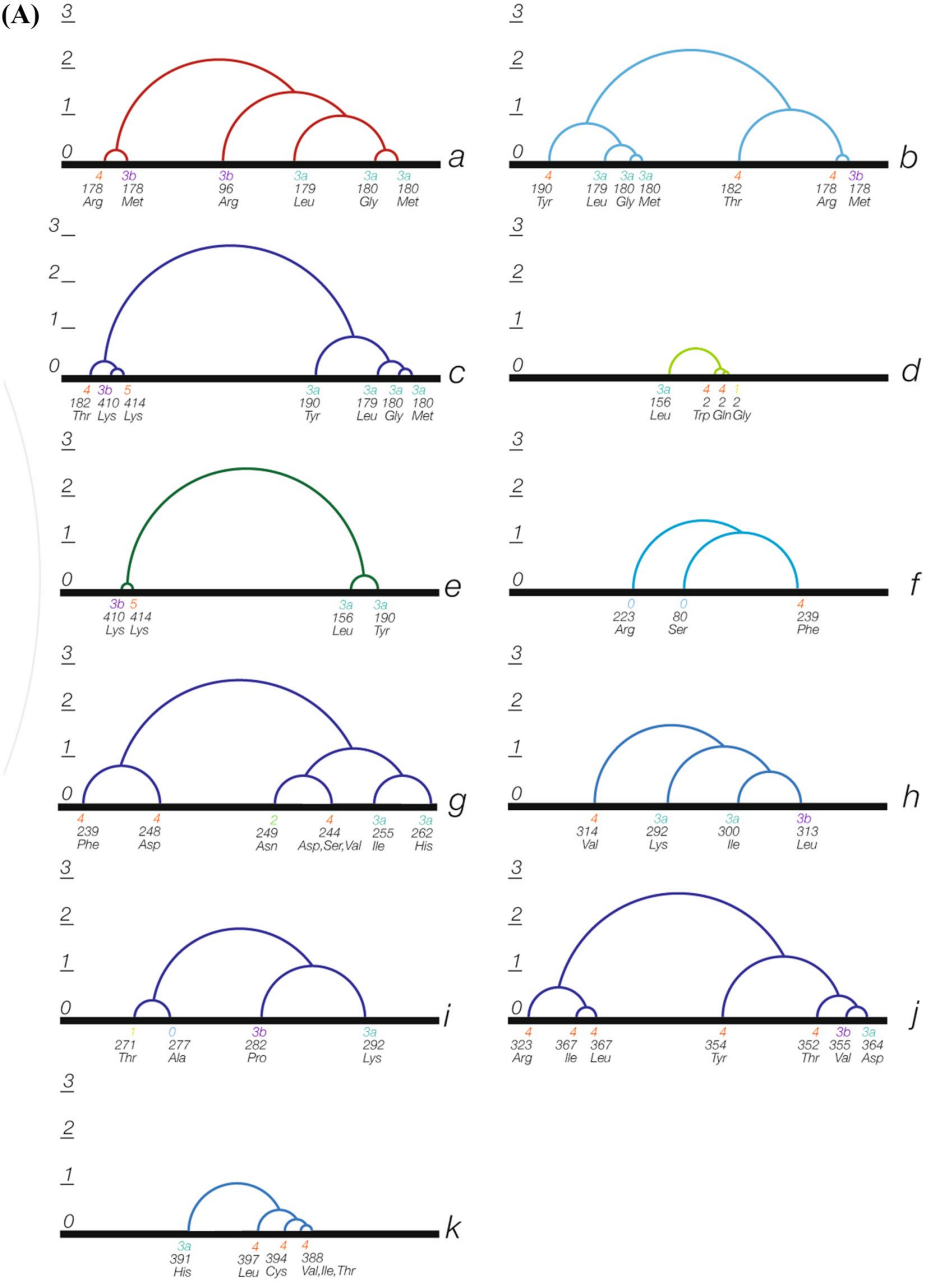

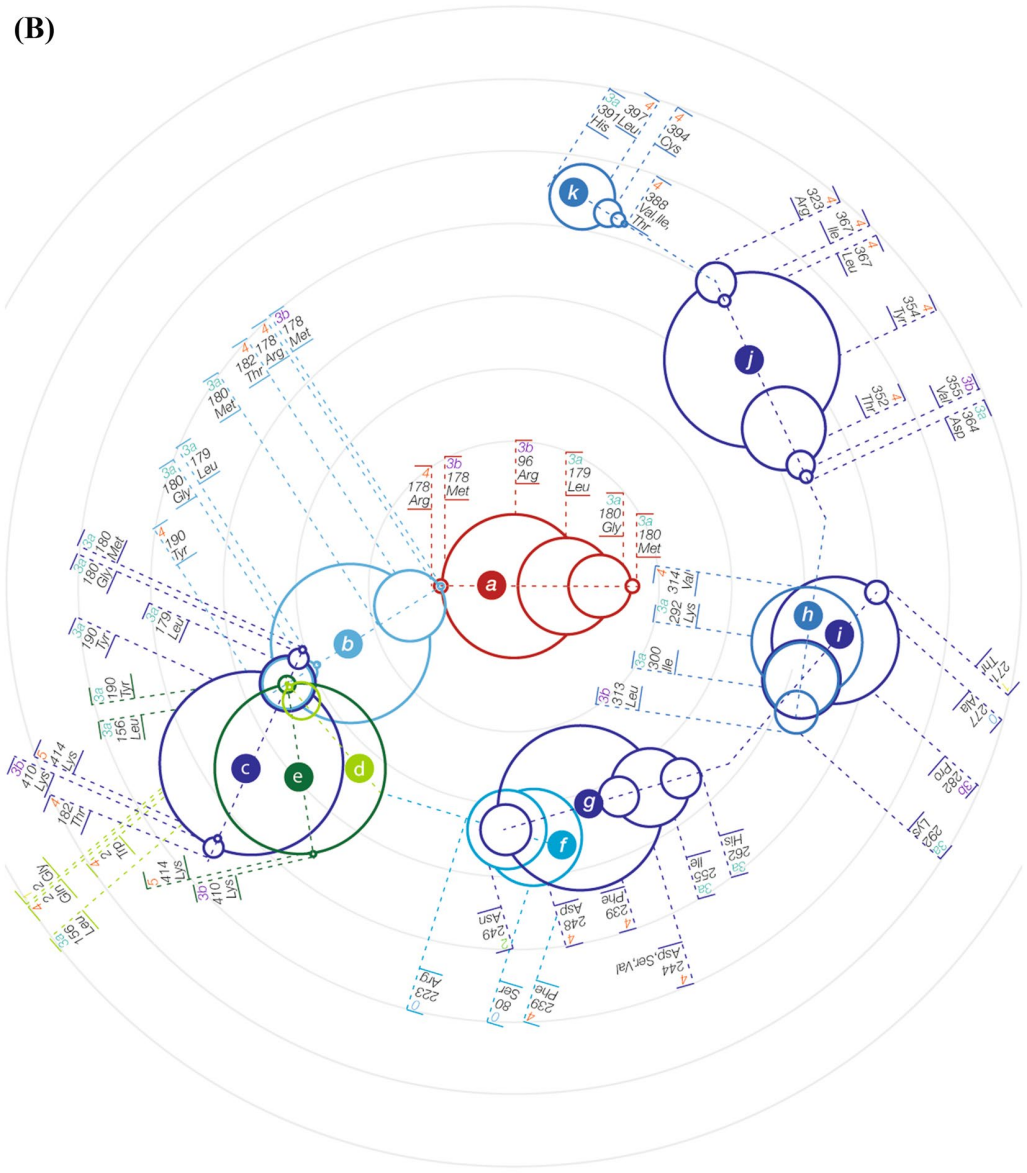
Normal mode analysis results for the analyzed *TUBB4A* variants. ΔG cluster analysis results are shown in dendrograms of the eleven identified structural clusters of variants (marked a–k). Each variant’s phenotypic subgroup is indicated next to it (according to the phenotypic subgrouping detailed in Table 1: subgroups 1, 2, 3a, 3b, 4 and 5. Subgroup 0 includes benign/likely benign variants from ClinVar and the p.Gly277Ala variant from GnomAD). (**A**) Separate standard dendrograms. ΔG distances between subgroups of variants in each structural cluster are represented by the corresponding cross-correlation coefficient differences in the Y-axis. Variants in each dendrogram are ordered in the X-axis according to their relative ΔG distance from the other variants in the corresponding structural cluster. (**B**) The eleven dendrograms (marked a–k) are shown with their shared variants and transition points. Variants shared by more than one structural cluster are shown in all their corresponding dendrograms. Each dendrogram is represented by a group of circles sharing a common axis through their diameters. The common axis of each of the dendrograms is marked by a dashed line and the variants in each corresponding structural cluster are indicated by lines perpendicular to that axis. Relative ΔG differences between subgroups of variants in each structural cluster are represented by the lengths of circles’ radii. The variants are ordered in each dendrogram according to their relative ΔG distance from the other variants in the corresponding structural cluster.

Investigation of potential genotype–phenotype correlation based solely on the position of each variant in the 3D protein model did not yield a significant correlation (Fig. 2A). While variants in several positions do correlate with a specific phenotypic subgroup, others in close Euclidean proximity to these variants are associated with a significantly different clinical consequence. Furthermore, when using only the calculated allosteric NMA thermodynamic fluctuations of each variant, we noticed only a partial correlation with the clinical phenotypic subgroup (Fig. 2B). On the other hand, a combination of both Euclidean (positional) and thermodynamic (entropic) profiles of each variant revealed a partial structural local (Euclidean) dependance between each variant and the clinical consequence, as the allosteric thermodynamical fluctuations showed a unique correlation pattern in each local area of the structure. Thus, we first identified eleven partially overlapping structural clusters (hotspots) of the variants (Fig. 3, clusters a through k) to be used as the basis for the NMA described further on. The mean distances between all variants in each of these clusters are shown in Fig. 3A, the relative distances between the corresponding center of mass of each of the clusters (centers A through K) are shown in Fig. 3B, and the positions of the analyzed *TUBB4A* variants in the 3D protein structure are shown in Fig. 3C. Transition points, in which two structural clusters shared one or several overlapping variants, were found between clusters a and b, b and c, b and e, c and e, d and e, f and g, and h and i, as demonstrated in Fig. 4.

### NMA results reveal genotype–phenotype correlation

Each of the above-mentioned structural clusters included variants of at least two types of phenotypic subgroups, negating investigation of potential genotype–phenotype correlation based on structural clustering only. Plots of the entropic profiles (ΔG) obtained by NMA for each of the analyzed variants are shown in Supplementary Fig. 2. We identified a unique pattern correlating the entropic profile with the phenotypic subgroup. For each subunit in the protein structure (A, C, D, E and K) we noticed a unique pattern of correlation. Notably, some of the clusters had almost identical patterns over several subunits while others showed different patterns in different subunits of the structure. Yet, all eleven structural clusters had at least one subunit affected allosterically in such a way that yielded a correlation of the entropic profile gradient with the phenotypic subgroup gradient, as described hereinafter.

We analyzed the NMA results for each structural cluster (hotspot) separately, using ΔG distances-based clustering of the variants in each of the structural clusters and ordering them based on their relative ΔG cross-correlation distances from one another. This analysis revealed a continuum of genotype–phenotype correlation across each of the structural clusters. Figure 4 shows these results in detail for all the variants analyzed. The results for several of these variants are elaborated here to highlight the main findings and their potential implications.

#### NMA-based differentiation of variants that impact the same amino acid position

Notable examples are the variants resulting in changes at amino acid position 2. Two of the three pathogenic variants affecting this position of the protein (p.Arg2Trp and p.Arg2Gln) are associated with the severe c-H-ABC phenotype, while the third (p.Arg2Gly) is associated with a different phenotype, DYT-4 dystonia (phenotypic groups 4 and 1 in Table 1, respectively). Correspondingly, NMA-based clustering showed that ΔG difference between p.Arg2Trp and p.Arg2Gln was smaller than the difference between p.Arg2Trp and p.Arg2Gly, as shown in Fig. 4. Furthermore, ΔG cross-correlation-based cluster analysis of the variants in structural cluster-d indicated that all three changes at position 2 are clearly separated from the p.Arg156Leu variant, which is associated with phenotypic subgroup 3a. These NMA-based relative ΔG cross-correlation distances between the four variants in structural cluster-d correlate with their associated phenotypes, yielding a genotype–phenotype continuum across this cluster. This finding demonstrates the potential prediction of phenotype by the NMA-based model, even for different variants affecting the same amino acid position.

#### NMA-based differentiation of the p.Asp249Asn variant

Additional analysis results of note are those of p.Asp249Asn, the most common pathogenic *TUBB4A* variant, which is associated with a milder H-ABC course^2,3^. Accordingly, this variant was included in the analysis as a separate phenotypic group (m-H-ABC; phenotypic group 2 in Table 1). As shown in Fig. 4, ΔG cross-correlation-based cluster analysis of the variants in structural cluster-g yielded a notable separation of this variant from the other pathogenic variants in this structural cluster which are associated with other *TUBB4A* phenotypic subgroups, including the ones associated with the more severe H-ABC phenotype (c-H-ABC; phenotypic group 4 in Table 1). This finding clearly demonstrates that the NMA-based model we applied enables entropy-derived differentiation of variants associated with different phenotypic subgroups.

#### NMA-based differentiation of control variants

Control *TUBB4A* variants, which are not associated with disease (benign, likely benign and common variants), were clearly separated by the NMA-based analysis from the pathogenic ones. This is in accordance with the entropy-derived genotype–phenotype continuum found across structural clusters. ΔG-based cluster analysis of the variants in structural cluster-i disclosed that p.Ala271Thr, which is associated with phenotypic subgroup 1 (the milder DYT-4 dystonia phenotype), and the common GnomAD variant p.Gly277Ala, were clearly separated from variants p.Glu292Lys and p.Arg282Pro, which are associated with phenotypic subgroups 3a and 3b, respectively (dendrogram-i in Fig. 4). Similarly, in structural cluster-f, the ΔG difference between the control variants p.Pro80Ser (benign) and p.Gly223Arg (likely benign) was smaller than the difference between p.Gly223Arg and the third variant in this structural cluster, p.Cys239Phe, which is a pathogenic variant associated with phenotypic subgroup 4 (c-H-ABC in Table 1). This entropy-based order of the variants in structural cluster-f was in line with the genotype–phenotype continuum characterizing the other structural clusters. Yet, the small number of variants in this structural cluster limited the investigation of the genotype–phenotype transition from p.Pro80Ser to p.Cys239Phe (dendrogram-f in Fig. 4).

#### Continuum of genotype–phenotype correlation in transition areas shared by different structural clusters

The continuum of genotype–phenotype correlation based on ΔG cluster analysis, was kept also in most of the transition areas where variants are shared by different structural clusters. This finding further supports the NMA-based tool we utilized to predict genotype–phenotype associations in *TUBB4A*-related disorders (Fig. 4B). Nevertheless, this continuum was interrupted in two transition areas between structural clusters. The first is the overlap area between clusters a and b, which includes the variants p.Thr178Met and p.Thr178Arg, associated with phenotypic subgroups 3b and 4, respectively. The second is the transition area between clusters c and e, which includes the variants p.Glu410Lys and p.Asn414Lys, associated with phenotypic subgroups 3b and 5, respectively. In both these instances, the two variants are associated with different phenotypic subgroups although the ΔG difference between them is relatively small. These findings suggest that in these transitional areas there may be additional more predominant structural factors, as well as other variables, that affect the phenotypic consequence of these variants, overriding the effect measured by the entropy changes on which our analysis was based.

## Discussion

Variant interpretation and investigation of genotype–phenotype correlation constitute major challenges in clinical genetics. Many in-silico methods have been developed as part of the effort to overcome these challenges. Traditional computational techniques to study the effect of variants on protein structure are heavily based on protein sequence data analysis and do not consider the protein’s intrinsic movements^7^. Therefore, much of the effect of genetic variants on the protein function is missed when using these approaches. The great progress in the characterization of the 3D structures of proteins lead to the development of new in-silico functional tools to improve genetic variant interpretation^40^. Newer in-silico approaches combine protein structure and dynamics to investigate the impact of genetic variants on the protein stability. In this study we used an NMA-based in-silico tool, which provides representation of intramolecular interactions, thereby representing more accurately the conformational changes in terms of calculated squared overlap. This approach introduces dynamic aspects of the protein into variant analysis. It was recently used successfully to categorize missense variants according to their effect on entropy^41,42^. We demonstrate the applicability of an entropy-based in-silico NMA tool in the investigation of genotype–phenotype correlation and its potential use in variant interpretation and phenotype prediction in *TUBB4A*-related disease.

### Position-only, entropy-only and combined position-entropy approaches

We found that a method based exclusively on either the variant’s position or the NMA-based thermodynamic fluctuations, yielded only partial or no variant-phenotype correlation. This finding lead us to combine the variant’s positional and entropic profiles by identifying eleven partially overlapping structural clusters of variants and analyzing each of these clusters separately, an approach which yielded a clear genotype–phenotype correlation. This finding shows that neither the variant’s position nor the entropic profile can be used separately and that NMA-based investigation of *TUBB4A* variants is best utilized when analyzing variants impacting amino acid positions in relative proximity to one another in the 3D protein structure. Moreover, these results indicate that additional factors other than NMA-related ones have a greater impact on the phenotypic consequence when considering protein variants located in greater physical distance from one another (i.e. variants not shared by the same structural cluster). Yet, these additional factors are not purely positional in nature, as our attempt to analyze *TUBB4A* variants based strictly on the position of each variant in the 3D protein model did not yield a significant genotype–phenotype correlation.

### NMA results support genotype–phenotype correlation and can aid in phenotype prediction in *TUBB4A*-related disease

Previous studies have reported the correlation between several *TUBB4A* pathogenic variants and their associated phenotype. At the less severe end of the phenotypic spectrum, the variants p.Arg2Gly and p.Ala271Thr are associated with DYT4, an adult-onset isolated dystonia^4,5^. At the most severe end of the spectrum, the p.Asn414Lys is associated with a severe infantile-onset presentation, characterized by lack of brain myelination, cerebellar atrophy and preserved basal ganglia on neuroimaging and severe encephalopathy with early death^2,6^. Between these two ends of the phenotypic spectrum, *TUBB4A*-related disease includes hypomyelination with atrophy of the basal ganglia and cerebellum (H-ABC), as well as cases of hypomyelination with no basal ganglia atrophy, both with different degrees of severity (Table 1). Of note, the most common *TUBB4A* pathogenic variant, p.Asp249Asn, is associated with a slower progression of the H-ABC phenotype^3^. Based on these data, a potential disease continuum associated with *TUBB4A* pathogenic variants has been previously suggested^2,3^. Yet, this suggestion was based on specific groups of *TUBB4A* variants and was not shown to apply for all reported disease-associated variants in this gene. The entropy-based in-silico NMA performed in this study revealed a continuum of genotype–phenotype correlations across each of the analyzed structural clusters of known pathogenic *TUBB4A* variants. Our results indicate that the higher the correlation of relative entropy difference between two variants in the same structural cluster, the higher is the likelihood these variants are associated with a similar phenotype. The previously suggested genotype–phenotype correlation for some *TUBB4A* variants is further supported by our findings, which extend this correlation to include all reported disease-associated variants in this gene, demonstrating a continuum of genotype–phenotype correlation across the structure of the encoded protein.

Notably, our analysis yielded genotype–phenotype correlation even for *TUBB4A* variants that are associated with conspicuously diverse clinical consequences although impacting the same amino acid position, as shown by the NMA results for variant substitutions of the Arginine at position 2 of the protein. It was previously suggested that the change of Arg2 to the relatively inert Glycine may have a limited effect on the protein function compared to the variants substituting the positively charged Arg2 either with the larger polar Glutamine or the hydrophobic Tryptophan, which are likely to have a substantial impact on the protein structure, potentially accounting for the striking difference between the clinical outcome associated with p.Arg2Gln and p.Arg2Trp (c-H-ABC) and the one associated with p.Arg2Gly (DYT4 dystonia)^3^. Our results further support a structural impact as the possible explanation for this phenotypic difference, while adding considerations of protein dynamics to the potential mechanism that may explain this observation.

As previously mentioned, a well-established genotype–phenotype correlation is the one associated with the most common pathogenic *TUBB4A* variant, p.Asp249Asn. This variant results in a relatively less severe disease course compared to other variants associated with the H-ABC phenotype^3^. Our NMA results support this genotype–phenotype correlation, as they clearly separate this variant from other variants in the same structural cluster, including ones associated with a more severe course of H-ABC (Table 1, Fig. 4). Furthermore, considering that the genotype–phenotype correlation associated with the p.Asp249Asn variant is well-established, our results strongly support the potential of the approach used in this study to assist in phenotype prediction in *TUBB4A*-related disease.

In summary, the previously suggested genotype–phenotype correlation in *TUBB4A*-realted disease is supported by our NMA results, which extend this correlation to all disease-associated variants across the encoded protein. Furthermore, since our results yield a clear genotype–phenotype continuum across each structural cluster, the in-silico approach we used can aid in phenotype prediction for additional *TUBB4A* variants, based on their incorporation into this NMA-based continuum. Therefore, our results support the integration of the approach used in this study in the investigation of genotype–phenotype correlation and phenotype prediction in *TUBB4A*-related disease.

### Application of NMA in the investigation of *TUBB4A* variant pathogenicity

The results of this study indicate the potential applicability of our methodology not only in the investigation of genotype–phenotype correlation, but also in the interpretation of pathogenicity of additional *TUBB4A* variants. The interpretation of the multitude of variants identified in the next-generation sequencing era is based on a combination of several parameters, including the variant’s frequency in the population, phylogenetic conservation, relevance of the gene to the phenotype, segregation of the variant in the pedigree, as well as information from different databases regarding the sequence alteration. In addition, different computational prediction tools are used to evaluate the expected impact of missense variants on protein function, and guidelines for the interpretation of sequence variants have been issued^43^. Nevertheless, variant interpretation remains limited and potentially inaccurate. Moreover, the number of missense variants classified as ones of uncertain significance (VUS) is increasing^40,44^. The gold standard evaluation of genetic variants is based on functional studies. Yet, these studies require considerable resources and time to be invested for each single variant investigated. Therefore, additional reliable and efficient tools are needed in order to improve variant interpretation in clinical genetics. Our ΔG-based cluster analysis showed that *TUBB4A* variants not associated with disease (control variants) were integrated into the genotype–phenotype continuum as expected and were clearly separated from pathogenic variants. This finding supports the potential contribution of our in-silico approach to the investigation of pathogenicity of additional *TUBB4A* variants. Moreover, our results support the potential implementation of the methodology that was used in this study for missense variant interpretation in additional monogenic conditions. This should be further investigated in future studies.

### Limitations

This study has several limitations. First, the number of analyzed *TUBB4A* pathogenic variants was small, as our search yielded only 41 such variants. The limited number of variants results in gaps in the information obtained by our analysis for each of the structural clusters identified, as well as for the parts of the protein that are not included in any of these clusters. Yet, we demonstrated a genotype–phenotype continuum across the informative parts, despite the small number of variants available for analysis.

Second, most of the *TUBB4A* pathogenic variants included in our analysis (25/41, 61.0%) are private mutations (Table 1). This may yield potential inaccuracies in the phenotypic variant subgrouping our analysis was based on, as the phenotypic information available for many of the variants was based on single case reports. Nevertheless, each of the variants reported in multiple cases (including the most common disease associated *TUBB4A* variant p.Asp249Asn, among others) is associated with the same phenotypic subgroup in all its associated cases (Table 1) and had NMA results which were in line with the ΔG-based genotype–phenotype continuum revealed by our analysis. Incorporation of additional pathogenic *TUBB4A* variants and phenotypic data into the entropy-based model we used will enable further refinement of this model and will improve the investigation of variant pathogenicity and phenotype prediction with this in-silico tool.

Third, the phenotypic consequence of a pathogenic variant may be affected by additional structural variables as well as other factors, such as modifier genes. These additional factors may have a more predominant impact on the clinical consequence than the effect measured by the entropy change which was used as the basis for our analysis. This may limit phenotype prediction by our model, as exemplified in our study in the transition areas between clusters a and b and between clusters c and e. In this regard, variant interpretation should not be based solely on the approach presented in this study and should take into consideration additional bioinformatic data. Nevertheless, our results were consistent for the vast majority of the analyzed variants. Therefore, we propose the integration of the NMA-based approach that was used in this study into variant analysis, since it contributes invaluable information regarding variant pathogenicity and genotype–phenotype correlations, as demonstrated by our results.

Finally, it is notable that the NMA-based model we used in this study is appropriate only for missense variant analysis, negating its use for other types of genetic variants. However, all reported pathogenic *TUBB4A* variants reported thus far are missense variants, enabling an effective use of this model in *TUBB4A*-related disease. Moreover, as missense variants are more common than other variant types^44,45^ and their interpretation constitutes a pivotal challenge in clinical genetics^40^, our tool may have a significant contribution to variant interpretation and phenotype prediction in other monogenic conditions.

### Future directions

Newer in-silico approaches that were developed in recent years exploit the availability of 3D protein structures to add the dynamic features of proteins to computational prediction tools^7^. To our knowledge, our study is the first to integrate protein dynamics into the investigation of genotype–phenotype correlation and variant pathogenicity in *TUBB4A*-related disease. The NMA-based genotype–phenotype continuum we found in this study should be updated periodically in the future to include additional *TUBB4A* variants and clinical data that will be reported further on. Additional data incorporated into this model will allow a more accurate investigation of *TUBB4A* variants by this tool. Future investigation of additional monogenic disorders with the NMA-based methodology we used in this study may further establish this approach as a tool for variant interpretation and phenotype prediction. Other future directions may include the application of additional approaches, other than NMA, that exploit protein dynamics in variant interpretation. This could be investigated in *TUBB4A*-related disease as well as in other monogenic conditions. Additional such approaches may include, for example, protein dynamics simulations^7^. Moreover, comparing the results obtained by different protein dynamics-based approaches may shed light on the advantages and disadvantages of each of these approaches in *TUBB4A*-related disease and in additional monogenic disorders. Furthermore, we speculate that a combined approach using a multitude of tools that exploit protein dynamics may have a great contribution to phenotype prediction and variant interpretation in clinical genetics. This is a subject for further investigation in the future.

## Conclusion

In this study we used an NMA-based in-silico tool to investigate genotype–phenotype correlations in *TUBB4A*-related disease, thereby integrating protein dynamics in variant investigation. We demonstrate a continuum of genotype–phenotype correlation across each of the structural clusters analyzed. Our results support genotype–phenotype correlation in *TUBB4A*-related disease. Moreover, they suggest that application of the approach used in this study will aid in the interpretation of variant pathogenicity and in phenotype prediction in patients carrying *TUBB4A* variants. Additional *TUBB4A* pathogenic variants and phenotypic data that will be added to this model in the future will enable to improve and refine its use. Furthermore, our results suggest the potential utilization of the methodology used in this study in the investigation of variant interpretation and phenotype prediction in additional monogenic conditions.

## Supplementary Information

Supplementary Legends.

Supplementary Figure 1.

Supplementary Figure 2.

## Data Availability

All data generated or analyzed during this study are included in this published article.
