# Supplementary material for: In-silico phenotype prediction by normal mode variant analysis in TUBB4A-related disease
Source: Sci Rep. 2022 Jan 7;12:58. doi: 10.1038/s41598-021-04337-x (PMC8741991; doi:10.1038/s41598-021-04337-x)
Supplement: Supplementary file 1 — Supplementary Legends. [file 41598_2021_4337_MOESM1_ESM.docx]

**Supplementary figure 1. Comparing different protein modeling approaches by Ramachandran plots.** First row: Ramachandran plots of the crystallographic structure of chains C (upper left panel) and B (upper right panel) in the Tubulin:Stathmin-like domain complex (1FFX). Second row: Ramachandran plots of the 1FFX chains C (middle left panel) and B (middle right panel) following PyMol mutagenesis to agree with the amino acid sequence of the human TUBB4A protein. Third row: Ramachandran plots using homology modeling by the Swiss model server of the original 1FFX chains C and B to human Tubulin alpha-1A (lower left panel) and human TUBB4A (lower right panel), respectively.

**Supplementary figure 2. Entropic profile (ΔG) of each of the analyzed variants using Normal Mode Analysis**. ΔG was computed as G_variant_-G_WT_, as described in the methods part. In each plot, the overlayed faded color plot indicates ΔG with an increase by a factor of 2000 to highlight details of ΔG. The color of each variant plot matches the color of the variant’s phenotypic subgroup labeling in Fig. 4.
