# Supplementary figures and images for: In-silico phenotype prediction by normal mode variant analysis in TUBB4A-related disease
Source: Sci Rep. 2022 Jan 7;12:58. doi: 10.1038/s41598-021-04337-x (PMC8741991; doi:10.1038/s41598-021-04337-x)

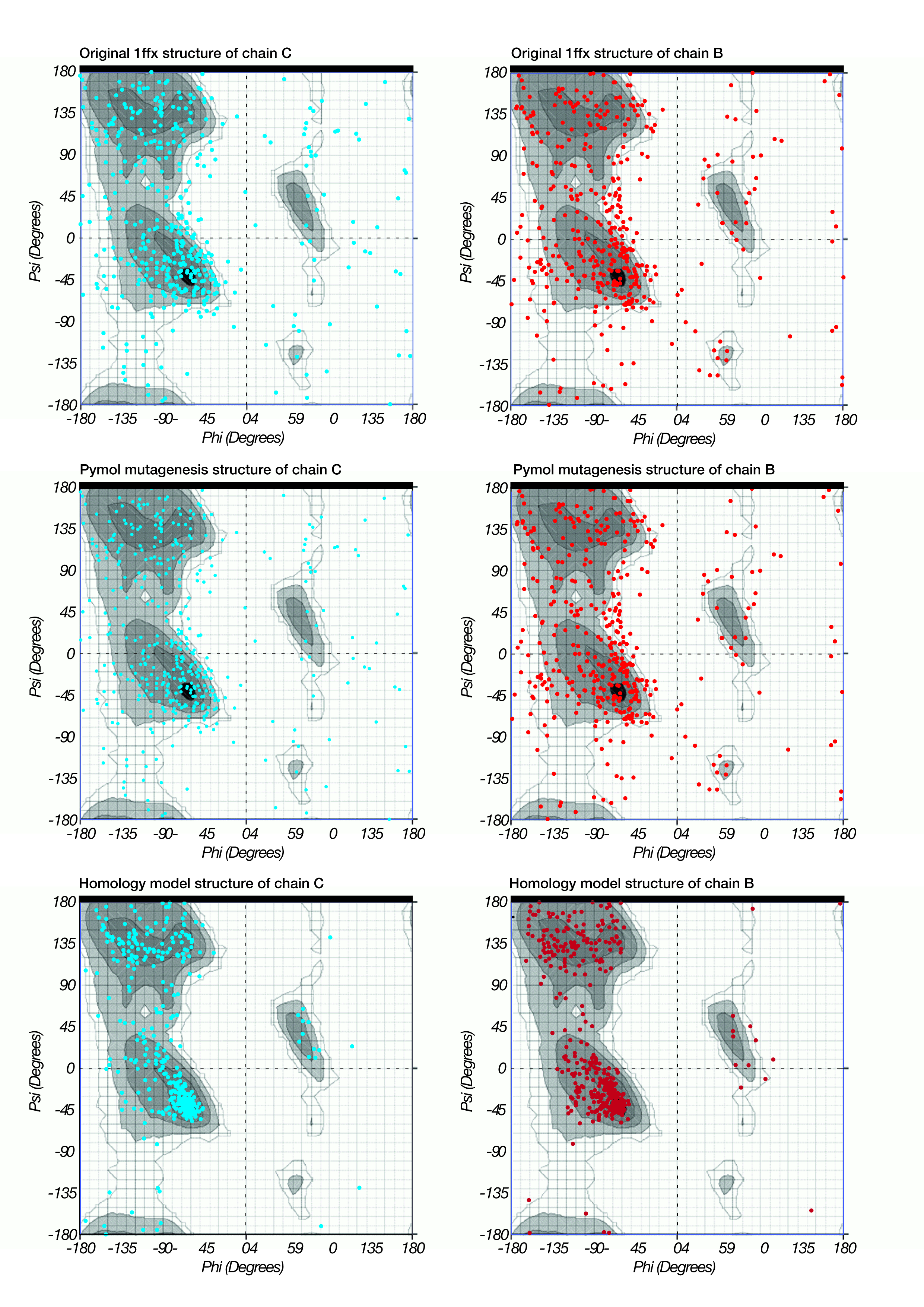

Supplement: Supplementary file 2 — Supplementary Figure 1. [file 41598_2021_4337_MOESM2_ESM.tif]

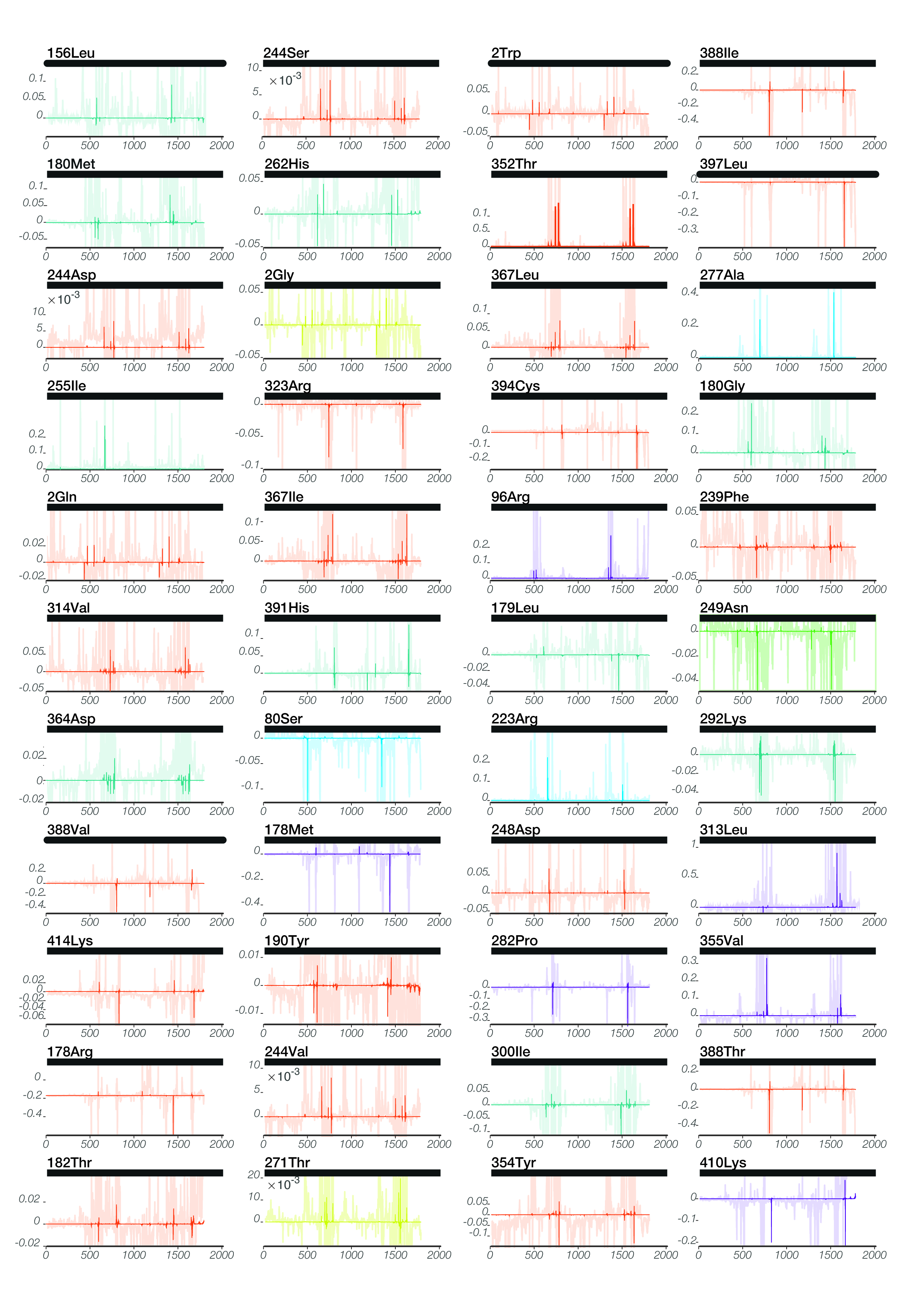

Supplement: Supplementary file 3 — Supplementary Figure 2. [file 41598_2021_4337_MOESM3_ESM.tif]
